# Interpenetration Networked Polyimide–Epoxy Copolymer under Kinetic and Thermodynamic Control for Anticorrosion Coating

**DOI:** 10.3390/polym15010243

**Published:** 2023-01-03

**Authors:** Dong-Sen Chen, Chun-Hua Chen, Wha-Tzong Whang, Chun-Wei Su

**Affiliations:** 1 Department of Materials Science and Engineering, National Yang Ming Chiao Tung University, Hsinchu 300, Taiwan; 2 Material and Chemical Research Laboratories, Industrial Technology Research Institute, Hsinchu 310, Taiwan

**Keywords:** polyimide, epoxy, anticorrosion, interpenetration, microphase separation, kinetic vs. thermodynamic control

## Abstract

Epoxy (EP) was copolymerized with polyamic acid (PAA, precursor of polyimide (PI)) with termanil monomers of (1) 4,4′-Oxydianiline (ODA) and (2) pyromellitic dianhydride (PMDA) individually to form (PI-O-EP) and (PI-P-EP) copolymers. The FTIR spectrum of PI-O-EP copolymerization intermediates shows that some amide-EP linkages were formed at low temperature and were broken at higher temperature; in additoin, the released amide was available for subsequent imidization to form PI. The curing and imidization of the amide groups on PAA were determined by reaction temperature (kinetic vs. thermodynamic control). In PI-P-EP, the released amide group was very short-lived (fast imidization) and was not observed on FTIR spectra. Formation and breakage of the amide-EP linkages is the key step for EP homopolymerization and formation of the interpenetration network. PI contributed in improving thermal durability and mechanical strength without compromising EP’s adhesion strength. Microphase separations were minimal at PI content less than 10 wt%. The copolymerization reaction in this study followed the “kinetic vs. thermodynamic control” principle. The copolymer has high potential for application in the field of higher-temperature anticorrosion.

## 1. Introduction

Metal corrosion, the unavoidable nature of chemistry, has long been a crucial problem to be resolved in many industries and has inflicted substantial economic loss to society’s economy. In fact, about one-third of corrosion hazards could be prevented and controlled using anticorrosion technologies, as addressed by the National Association of Corrosion Engineers (NACE) [1].

Nowadays, in highly populated areas, for various reasons, many industries are moving to places nearby the sea, where high concentrations of moisture and salt are major challenges. Even worse, operations occur at elevated temperatures, posing more severe corrosion damage to the equipment.

For years, organic coatings were used as a barrier that separates the metal and the corrosive environment to overcome the corrosion problems. The organic coatings were designed to selectively block out or at least slow down the diffusion of corrosive chemicals and thus protect the metal surface [2].

Epoxy (EP), exhibiting superb adhesive properties with metallic substrates, has long been recognized as an excellent corrosion-resistant coating material [3,4,5]. The strong adhesivity of EP with metals originates from its ring-opening reaction of the epoxide groups, but the downsides are lack of flexibility and low thermal stability. Various hardeners that participate in the epoxide ring-opening reactions have been comprehensively investigated, such as aliphatic or aromatic polyamines, polyamides, imidazole, dicyandiamide, carboxylic acids, anhydrides, and dianhydrides [6,7,8,9]. Among them, anhydride and dianhydride are considered good candidates for EP resin for providing desirable high cross-link density and high thermal durability. The above advantages could further be enhanced if the EP resin is cured with another high heat-resistant polymer with function groups that can also act as hardeners [3,10,11,12,13,14].

In the family of highly thermally stable polymer materials, polyimide (PI) is frequently chosen for industrial applications [15,16]. However, its low adhesion property has limited PI’s application as a coating material. Since PI was polymerized from aromatic dianhydride and aromatic diamines, the terminal groups could be either amine or anhydride. These two terminal groups of PI could serve as good functional groups in hardening EP resin [17,18,19,20].

Furthermore, in synthesizing PI, polyamic acid (PAA), the intermediate formed in the early stage, has both amide and carboxylic acid groups, which are the two main types of hardeners of EP. Therefore, the idea of combining the two and allowing simultaneous imidization (PAA to PI) and curing seems to be plausible [21,22,23,24,25]. A similar point of view is that the functional groups of EP and PAA could interact with each other. Therefore, compounding PI-EP to produce a copolymer could provide a solution to the above stated problems. Using PAA with different terminal functional groups could be a feasible way [26]. Xing et al. and others [27,28] used waterborne PI to modified waterborne EP resin by neutralizing the precursor (PAA) with 2-methylimidazol for PAA salt (PAAS) formation, after which the salt is basically waterborne. Accordingly, they found the copolymerized PAAS and EP copolymer with a semi-interpenetrating structure. They reported that the PAAS-EP copolymer has high cross-linking density and heat stability.

However, there is a concern that the amide groups on the PAA chain could easily react with EP, and if all or a major portion of them bonded during curing with EP, the obtained matrix would have very high crosslink density, which would result in high rigidity and becoming brittle; as a result, the matrix would not be suitable for coating purposes. Moreover, if the majority of amide groups on the PAA chain are cured with EP at a relatively low temperature, then there are fewer amide groups left for imidization to form PI, and the expected superior thermally stable PI-EP copolymer matrix will not be obtained. Previous studies [17,18,19,20,27,28,29,30] reported that the PI-EP copolymer matrix could be obtained as expected, and benefits from both PI and EP have been observed. There must be some critical points worth noticing in the process of copolymerization.

Microphase separation is yet another concern when making compatible two different polymers (physical blending or chemical linkage) [31,32]. Thus, avoiding or minimizing microphase separation must be emphasized. If a low-molecular-weight PI were used for minimizing microphase separation, the PI’s characteristic advantages in the copolymer would be minor or buried in the copolymer matrix. For EP coating, commonly an EP of equivalent weight (EEW) higher than 500 is used. For EP of higher EEW, the compatibility with PI tends to be low, and microphase separation is of concern. Hence, a low EEW of EP and a medium molecular weight of PI were used in this study.

Still, several questions remain unanswered and would be interesting to investigate. For example, how is each domain (PI and EP) established during the copolymerization process? Information on how the amide-EP linkage reaction and the competing imidization reaction are regulated, which determines the final structure and physical properties of the copolymer, is still needed. As for microphase separation, we need to know the why and how regarding whether it can be completely avoided or minimized.

## 2. Materials & Methods

### 2.1. Chemicals

The EP resin used in this study was a diglycidyl ether of bisphenol A (DGEBA) with EEW of 190, purchased from Shell Chemical, UK (Epon 828).

4,4′-Oxydianiline (ODA) with a purity of 98% was purchased from Criskev Company Inc. (Over Lake Park, KS, USA) and was used as received.

1,2,4,5-bezene tetracaboxylic dianhydride (pyromellitic dianhydride, PMDA) was purchased from Fluka (Charlotte, NC, USA). The purity of the PMDA was 98%. Before use, it was purified by recrystallization in acetic anhydride (4.5:1) followed by sublimation.

N-Methyl-2-Pyrrolidone (NMP) of 99% purity was obtained from Mallinchrodt Chemicals (St. Louis, MO, USA). The NMP was purified by distillation with a molecular sieve before use.

### 2.2. Preparation of Polyimide-EP Copolymer

Two types of PAA, namely PAA with terminal diamine and PAA with terminal dianhydride, were prepared. Before adding reagents to a three-neck flask, the flask was heated with constant nitrogen purge for one hour, and then the flask was allowed to cool with continued nitrogen purge to assure an oxygen-free condition [33].

In PI-O-EP preparation, oxydianiline (ODA, 0.24 mol, 48.10 g) was first dissolved in N-methylpyrrolidone (NMP) solvent by stirring (12 h). After complete dissolution of ODA, pyromellitic dianhydride (PMDA, 0.23 mol, 49.95 g) was added to the ODA solution. The mixture was stirred continuously for 12 h to obtain a homogeneous PAA solution of 15 wt%. Various weight ratios of EP resin were then added to the mixture and stirred in nitrogen for 3 h. The above mixture was then subject to thermal step imidization at various temperatures. Since a greater number of moles of ODA were added to the mixture, the resulting product was expected to be polyamic acid with terminal ODA, which later reacted with EP resin, and the resulting copolymer was termed PI-O-EP. The designed compositions of PI-O-EP copolymers are listed in Table 1.

In PI-P-EP preparation, ODA (0.24 mol, 48.10 g) was first dissolved in NMP solvent by stirring for 12 h. After complete dissolution of ODA, PMDA (0.25 mol, 54.30 g) was added to the ODA solution. Because an excessive number of moles of PMDA were used to prepare PAA, the terminal functional groups are dianhydride, which later react with EP resin; the resulting copolymer was termed PI-P-EP. The rest of the procedures are the same as in PI-O-EP preparation. The designed compositions of the PI-P-EP composite copolymer are also listed in Table 1.

### 2.3. Techniques Used in the Investigation of Synthesized Polymeric Materials

FTIR (ISF112V, Perkin-Elmer, Waltham, MA, USA) was used to monitor chemical changes in the characteristic function groups and to verify the progress of the designed chemical reactions. Reaction mixtures with the same composition were coated on separate salt chips (KBr) and baked in the same oven. At different temperatures, one was taken for FTIR analysis. The waveband range was 4000~500 cm^−1^. All samples were dried via 75 °C air blowing to remove residual solvent. The film thickness of each sample was controlled at 10 µm using a wire bar coater, as widely used in the ink and paint industry.

Thermal gravimetric analysis (TGA) was performed using a thermal analysis instrument (TGA-2050, TA Instruments, New Castle, DE, USA) in nitrogen with a heating rate of 20 °C/min.

Microscopic images of the fracture of the PI-EP copolymer were taken using a field emission scanning electron microscope (FESEM JEOL, JSM-6700F). Samples were coated with gold in vacuum on aluminum mounts with carbon paste. The survey mode was SE (secondary electron) imaging, the acceleration voltage was 3.0 kV and the electron light source was an in-lens Schottky field emission electron gun.

Corrosion resistances were evaluated using the salt spray test according to ISO standard 7253 [22]. Test specimens were placed in a closed chamber and exposed to a continuous indirect spray of 5% NaCl solution (pH range of 6.5 to 7.2). The chamber temperature was controlled at 35 °C. The corrosion resistance of the individual specimens was established on the basis of the difference in time before the first corrosion observed. (Salt Spray Tester, CHERNG JIA INSTRUMENT ENTERPRISE CO., LTD. SST-CB, Taiwan).

Tensile tests were performed according to ASTM D882 [34]. Specimens of 10 mm wide and 100 mm long were used for the test. Five samples were tested and the results were averaged. Each specimen’s elongation, tensile modulus, and tensile strength were analyzed by recording the tensile displacement and load, and the tensile rate was 10 mm/min. Tensile tests were conducted using a universal instrument (Gotech Testing Machines Inc. AI-7000-ST, Taiwan).

## 3. Results

### 3.1. Chemical Structures

Li. [35] commented that Raman spectroscopy and FTIR could be used to monitor the progress of corrosion on metal with an EP coating. In this research, we adopted the FTIR spectrum for verifying changes in featured function groups, mainly nitrogen-related amine and imide bonds as well as carboxyl groups.

**Figure 1:**
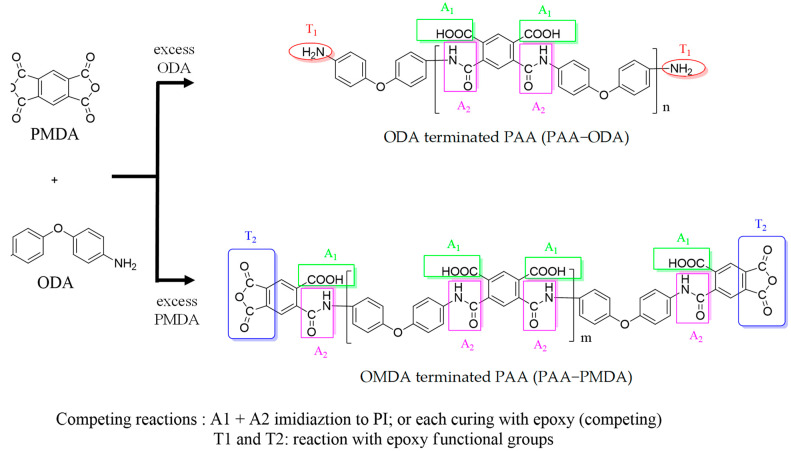
Reactive function groups of ODA terminated PAA (PAA-ODA) and PMDA terminated PAA (PAA-PMDA): amines (T1), anhydrides (T2), carboxylic acids (A1) and amides (A2).

In the first step of synthesizing PAA with diamine (PAA-ODA) and dianhydride (PAA-PMDA) as terminal function groups, PMDA was first allowed to react with ODA to form PAA in NMP solution. Excessive ODA was added to assure that the product was PAA with di-amine terminals. Alternatively, excessive PMDA was added for forming dianhydride-terminated PAA (Figure 1).

Since the solution was mixed thoroughly, the molecular weight (MW) of PAA could be controlled using the molar difference between ODA and PMDA. The reason why a medium MW of the PAA polymer is preferred over a large MW of PAA is that during subsequent reactions with EP, large molecules of PAA will likely lead to microphase separation during curing and imidization and make the copolymer unsuitable for coating applications.

**Figure 2:**
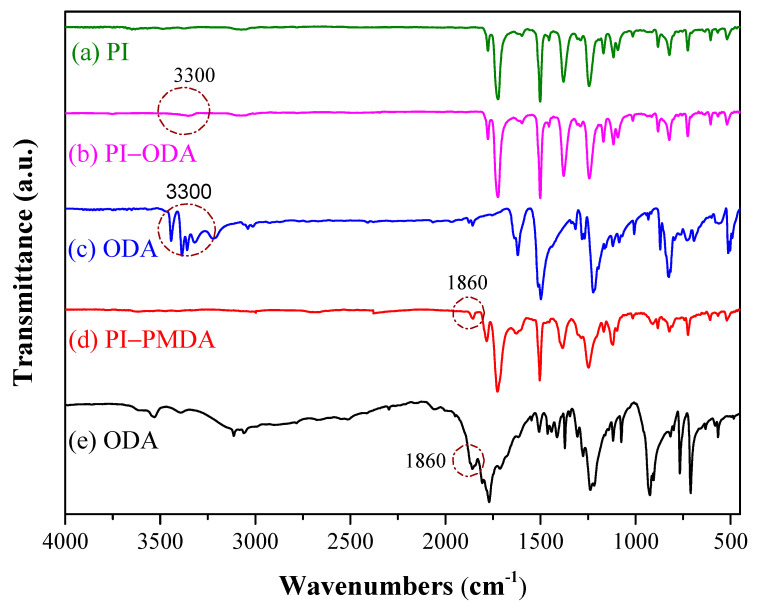
FTIR of (**a**) PI, (**b**) PIODA, (**c**) ODA, (**d**) PIPMDA, and (**e**) PMDA.

Figure 2 compares the FTIR spectrum of ODA, PI-ODA, and PI. In the FTIR absorption spectra of PI, the absorption peaks of C=O at 1720 cm^−1^ and a shoulder at 1777 cm^−1^ are characteristic for a PI polymer [36]. Meanwhile, the C-N (resulted from -NH_2_ reacting with anhydride) absorption at 1390 cm^−1^ was also observed. For the ODA spectra, the diamine (N-H) function group’s absorption peak was observed at 3200–3300 cm^−1^.

Comparing the PI-ODA spectra with that of PI (Figure 2a,b), the characteristic C=O absorption (1720 and 1777 cm^−1^) and the C-N absorption at 1390 cm^−1^ are almost identical. In the spectra of PI-ODA, the N-H absorption peak is weak but observable. This indicates that the MW of PI-ODA was not large enough to make the N-H signal unobservable. The reason why PI-ODA, instead of PAA-ODA, is used to confirm the terminal function groups in the above discussions is that the absorption peak of carboxyl -OH (at 3500 cm^−1^) in PAA is broad and strong, which makes the terminal amine (3300 cm^−1^) absorption peak vaguely observable.

Figure 2 also compares the spectra of PI-PMDA to those of PI and PMDA. The featured C=O (1777 and 1720 cm^−1^) and C-N (1390 cm^−1^) absorptions of PI are observable. Unlike PI, the C=O absorptions in the PMDA spectra are broad (1860, 1806 and 1770 cm^−1^) due to the dianhydride carbonyl of PMDA. For the PI-PMDA spectra, there are two points worth noticing.

Firstly, the absorption peak at 1860 cm^−1^, which is characteristic of the C=O group in carboxylic dianhydride in PMDA, is absent in the PI spectra. This shows that in PI-PMDA, the anhydride group originating from PMDA remains observable. This would not be so if the MW of PI-PMDA was large, since a weak signal tends to be buried in strong signals attributed to large numbers of other function groups.

Secondly, the C=O absorption peaks at 1806 and 1770 cm^−1^ of PMDA are in close proximity to that in PI (1777 cm^−1^) and are overlapped (or merged) into the 1777 cm^−1^ peak (in the PI-PMDA spectra). This leads to a higher and broader peak at 1777 cm^−1^ compared to the 1720 cm^−1^ peak.

### 3.2. Reaction of PI-O-EP Formation

**Figure 3:**
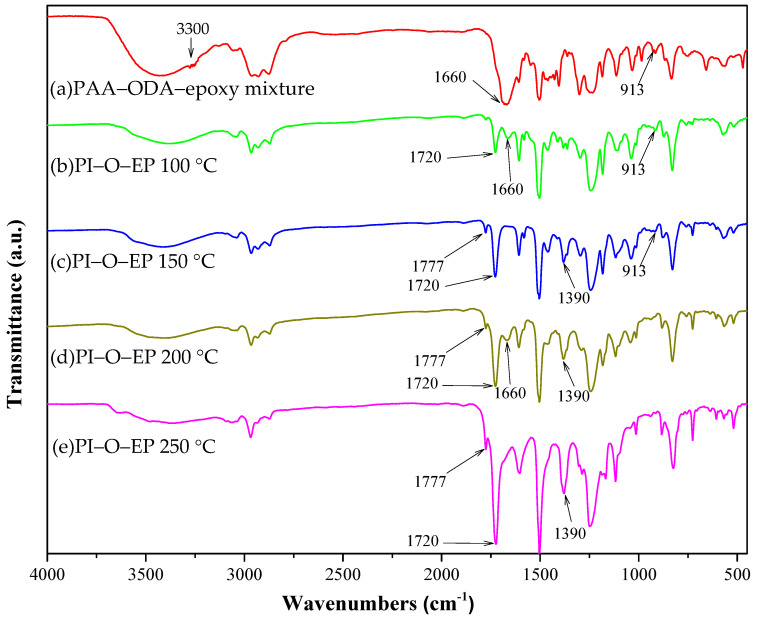
FTIR of the curing profile of PI(50)-O-EP(50) at different temperatures: (**a**) room temperature (unheated reaction), (**b**) 100 °C, (**c**) 150 °C, (**d**) 200 °C, and (**e**) 250 °C.

The purpose of presenting Figure 3 is to discuss the curing reaction of EP resin, where it is PAA that actually participates in curing, rather than PI (the reaction of PI formation competes with the curing reaction). In Figure 3, comparisons focus on four characteristic peaks: 3500 cm^−1^ (-OH, derived from carbonyl and epoxide ring opening), 1390 cm^−1^, 1777 cm^−1^ (imide C-N bond and C=O carbonyl absorption), and 913 cm^−1^ (C-O bond of epoxide ring). Meanwhile, Figure 1 shows that the functional groups (amine, carboxylic anhydride, carboxylic acid, amide) can react with EP or participate in imidization to form PI.

In the spectra of the PAA-ODA and EP resin mixture (the reactions with EP have not yet started), 3500 cm^−1^ corresponding to -OH (-COOH group of PAA) and terminal amine (3300 cm^−1^, appearing as a shoulder of the 3500 cm^−1^ peak) overlap into one broad and strong absorption peak. The peak at 3300 cm^−1^ disappears right after the reaction between the terminal amine and epoxide ring, which is fast at 100 °C [29]. Therefore, this overlapped shoulder at 3300 cm^−1^ diminishes soon after the curing reaction between the terminal amine and the epoxide ring.

Figure 3 reveals that when ODA terminated PAA (PAA-ODA) and EP resin mixture are allowed to react with EP at a 50%/50% weight ratio at a curing temperature of 100 °C (1 h), both the amide peak at 1660 cm^−1^ [23] and the acidic OH absorption peak (3500 cm^−1^) are strong. The disappeared 3200 to 3300 cm^−1^ shoulder peak indicates that the terminal diamine function groups reacted with epoxide rings. However, the C-N peak and C=O of PI (1390 and 1777 cm^−1^) are not observed. These indicates that at 100 °C, the imidization reaction has not begun yet. The epoxide ring peak (913 cm^−1^) is still salient, and most EP molecules still remain unreacted (at 100 °C).

At 150 °C (1 h), comparing the above five featured absorption peaks (3500, 1660, 1777, 1390, and 913 cm^−1^), possible chemical changes can be deduced as follows: The disappearance of the 1660 cm^−1^ amide peak [23], accompanied with the increase in the 1777 and 1390 cm^−1^ peaks (which is characteristic for PI) and the reduced 913 cm^−1^ peak, indicate that the amide group (1660 cm^−1^) is converted to imide in PI or amide-EP linkages. This indicates that some epoxide ring openings are possibly involved in the formation of nonterminal amide-EP and/or acid-EP linkages.

It is noteworthy that with increasing temperature (100–150 °C), the intensity of the OH absorption peak (3500 cm^−1^) remains nearly unchanged or slightly increased, which may appear to contradict the expectation that the amount of acidic OH will be reduced during imidization. This can be explained as follows

**Figure 4:**
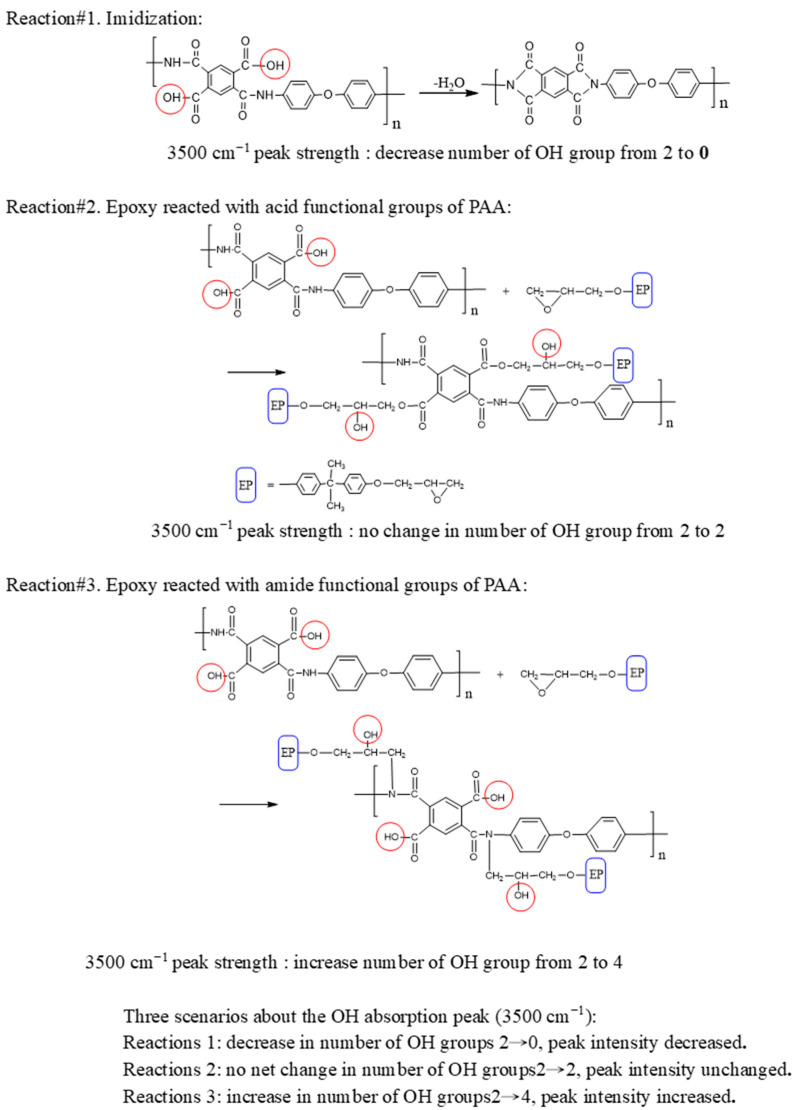
Reactions of acid or amide function groups of PAA with EP, as well as imidization reaction.

During imidization of PAA-O-EP to PI-O-EP, which accompanies the curing reaction with EP, the process involves three possible reactions: (1) imidization, (2) acid-EP linkage formation, and (3) amide-EP linkage formation (Figure 4). In the imidization reaction (reaction #1), an amide group reacts with a carboxylic acid group. When forming an imide bond, a water molecule evaporates, which leads to the loss of one N-H and one OH group. This reaction leads to reduction in the total number of OH groups and consequently decreases the 3500 cm^−1^ peak’s intensity. However, there is no decrease in peak intensity at 3500 cm^−1^, which reveals that imidization has not yet begun, or has happened only to a small extent at these temperatures. It is consistent with PI fabrication that the imidization reaction occurs at a higher temperature, which implies a higher activation energy of the reaction.

In the reaction of acid-EP linkage formation, the carboxylic OH group reacts with an epoxide ring with ring opening while generating an alcoholic secondary OH. Hence, this reaction (reaction #2) does not cause change in the total number of OH groups and the 3500 cm^−1^ peak intensity is not affected (Figure 4).

In the amide-EP linkage formation (reaction #3), the amide nitrogen reacts with an epoxide ring with ring opening and generates an alcoholic secondary OH on an EP segment. Therefore, reaction #3 increases the total number of OH groups; hence, the 3500 cm^−1^ peak strength should increase accordingly. If reaction #3 (3500 cm^−1^ OH peak increased) occurs or is accompanied by reaction#1 (3500 cm^−1^ OH peak decreased) and they are taking place simultaneously, the net effect would be unchanged or slightly increased peak intensity at 3500 cm^−1^ at 150 °C. Comparing the effects of the above three reactions, the amide-EP linkage reaction (reaction #3) or reaction #3 accompanied by imidization (reaction #1) are dominant during curing. The above inference is consistent with the reactivity of the EP curing reaction, where the amide function group is another reactive group with an epoxide ring (but is less reactive than amino group), the activation energy of EP-amide curing should be low, and therefore it is easier for the curing reaction to take place at medium temperature.

The 1660 cm^−1^ amide peak reappears in the 200 °C spectra, indicating that some of the nonterminal amide-EP linkages were broken and the amide-nitrogen was available for subsequent fast imidization reactions [29,30]. It is quite likely that the breakage of the amide-EP linkage was caused by the competing imidization reaction. The above observations follow the principles of reaction kinetics and thermodynamics of chemistry. Initially, at lower temperature (about 150 °C), some amide-EP linkages are formed (lower activation energy, kinetic control); at higher temperature, imidization is thermodynamically favored (more exothermic and stabled PI, thermodynamic control), and some amide-EP linkages are broken.

According to the principle of organic synthesis with regard to “kinetic vs. thermodynamic control”, which was used to explain the product distribution of the addition reaction of hydrobromide to 1,3-butadiene [37], at lower temperature, the major product was from the 1,2-addition reaction. Meanwhile, at higher temperature, the major product was from the 1,4-addition reaction. The reason of such difference lies in the differences of activation energies of these two reactions and the stabilities of the products. At lower temperature, the thermal energy was sufficient only to cross the activation energy for the 1,2-addition reaction (kinetic control), but was not high enough for the 1,4-addition reaction. At higher temperature, the thermal energy was high enough to cross the activation energies for both reactions. However, the product of the 1,4-addition reaction has lower potential energy, that is, the reaction product is highly stable; therefore, at higher temperature, the major product was from the 1,4-addition reaction (thermodynamic control).

Similarly, in the process of PAA-EP reactions to form PI-EP products, the curing of the amide-EP reaction and imidization reaction (of amide-acid to PI) could both be taking place, but the major reaction should be dependent on temperature (energy state), reactivity (activation energy), and product stability. In the process of forming PI-EP, as the temperature increases, there should be a kinetically controlled stage (lower temperature) and a thermodynamically controlled stage (higher temperature). This basic principle of organic chemistry is rarely discussed in regard to polymers, but can be applied here to explain certain phenomena observed in this study [38,39,40,41,42,43].

In the minireviews of J. Matern et al. [43], they discussed supramolecular polymerization (SP), where, quote, “in contrast to classical polymers, the presence of reversible noncovalent interactions” provides various functionalities in nature. In this article, a study by Ryu and Lee [44] was cited, where, quote, “the self-assembly of a bolaamphipilic molecule over a period of four weeks, the micellar kinetic assemblies transformed into the thermodynamically stable cylindrical architectures”. In this study, PI-EP copolymer is a classical polymer and allowed only covalent bonds in their matrix, which implies no reversible reaction. However, at different reaction temperatures or stages, because the system is not yet cooled down and is in a metastable state, the polymerization or curing reaction can be temporarily reversible. In supramolecular polymerization, the reaction (involving no change in covalent bond) can stay reversible for a long period of time. In contrast, in PI-EP polymerization, only for a short period of time during polymerization is the amide-EP covalent bond’s breaking and forming reversible.

During heating, the temperature was raised from room temperature to high imidization temperature (about 200–250 °C). At the early stage (about 150 °C), due to the high reactivities of the amide group, it could participate in the curing reaction with the epoxide ring of EP. Hence the amide-EP reaction would be dominant (kinetic control). Near the stage of high imidization temperature, the PI formation reaction would be dominant (thermodynamic control). In between, the amide-EP reaction could be considered reversible, since some amides were released for later imidization (PI forming) reaction, and the linked EP molecule was released as its diol form, which is important in inducing EP homopolymerization. This will be further discussed shortly in the IPN structure section.

From the viewpoint of the final copolymer matrix, PAA has abundant amide groups that can react with EP; if all or a major portion of these amide groups are bonded in curing with the epoxide ring of EP, the obtained matrix would have a very high crosslink density, which would result in high rigidity, becoming brittle; consequently, the matrix would not be suitable for coating purposes. Moreover, the PAA would not have enough amide groups for imidization to form PI, and the expected PI-EP matrix cannot be obtained, since the limited amount of PI is not able to contribute its superior thermal stability to the copolymer matrix. Therefore, at some intermediate point, the formed amide-EP bonding starts to break and release the amide function group and allow more opportunity for imidization.

Besides reacting with terminal amine, the epoxide ring may also react with amide or acid groups of PAA during curing, although the latter two reactions are substituted by the competing imidization reaction. Thus, PAA with terminal amines acts as a polymeric hardener of EP resin [29]. The subsequently formed PI is beneficial in rendering flexibility to the PI-EP copolymer. At the same time, PAA is in the process of imidization to form PI (1390 cm^−1^ and 1777 cm^−1^, imide C-N and C=O of carbonyl). According to Gaw et al. [29], the nonterminal amide-EP or acid-EP linkages are likely to break due to competitive imidization reactions. If so, the two -OH groups, generated by epoxide ring-opening and linkage breaking, further react with the epoxide ring of another EP molecule and lead to the homopolymerization of the EP resin. This mechanism implies that even with the breaking of amide-EP or acid-EP linkages, the inter-penetration structure is already established, which can provide the opportunity of combining both the desirable physical properties of PI and EP for the copolymer.

Upon the breakage of amide-EP linkage, the released EP molecule has already lost its epoxide function group, and the 913 cm^−1^ epoxide absorption peak does not recover accordingly. With an epoxide ring opened, thus-generated OH groups subsequently join the homopolymerization reactions of EP. Instead, the peak (913 cm^−1^) further decreases, which indicates that more EP molecules are consumed during the process. The residual, unreacted EP molecules may react with the OH groups on those released epoxides in the EP homopolymerization reaction [29].

In the 250 °C (2 h) spectra, the 1660 and 913 cm^−1^ peaks are not observable, indicating that the amide and EP molecules were fully reacted (imidization, terminal amine-EP, or homopolymerization of EP). The peak intensity at 3500 cm^−1^ is reduced, but still shows that some OH groups on the EP segment remain unreacted, which plays an essential role in the adhesion with metal.

### 3.3. Reaction of PI-P-EP Formation

**Figure 5:**
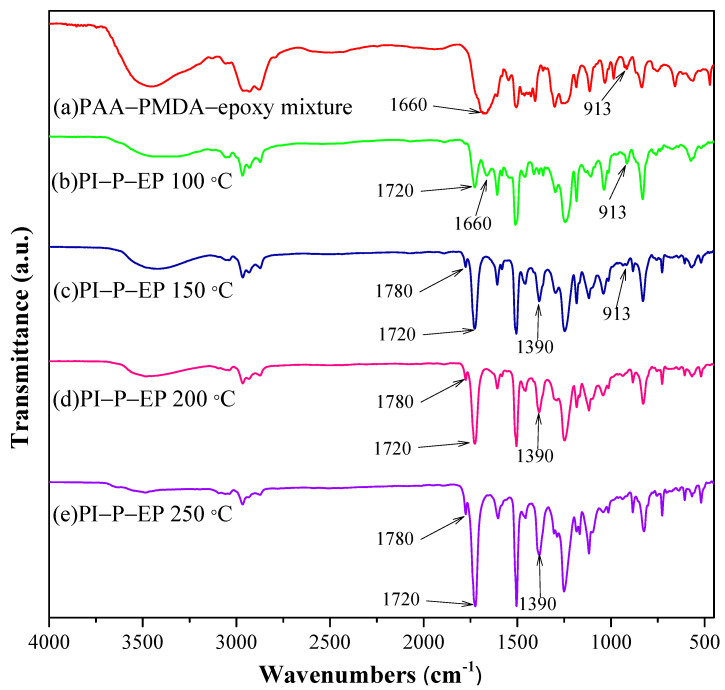
FTIR of the curing profile of PI(50)-P-EP(50) at different temperatures: (**a**) room temperature (**b**) 100 °C, (**c**) 150 °C, (**d**) 200 °C, and (**e**) 250 °C.

Figure 5a compares the FTIR spectrum of PAA with dianhydride terminal groups. In the spectra of PAA-PMDA (Figure 5b), the C-O absorption peak (of epoxide ring) can be observed at 913 cm^−1^, which indicates that epoxide ring-opening has not taken place yet (at 100 °C). This peak intensity begins to reduce above 100 °C, and continues to diminish to complete curing at 250 °C, which implies that a significant portion of the epoxide rings of the dosed EP molecules reacted with PI to form anhydride-EP linkages, and some EP homopolymerization occurred as well.

During the formation of PAA, excessive PMDA (Table 1) was used to ensure that the carboxylic dianhydride was the terminal group of PAA. The reactivity of EP with amide is faster than with carboxylic dianhydride or carboxylic acid. Assuming all terminal function groups are carboxylic dianhydride, the epoxide would first react with amide and then with carboxylic acid or dianhydride. However, once a non-terminal amide nitrogen has reacted with epoxide and formed a N-EP linkage, this nitrogen will not be able to participate in the imidization reaction during heating, unless the N-EP linkage is broken and releases a free amide that will immediately participate in imidization. Therefore, the free amide absorption peak at 1660 cm^−1^ is not observed again at 200 °C (as in Figure 5).

Figure 5 shows that the characteristic absorption peaks of PI, C=O (1777 and 1720 cm^−1^) grow stronger with increased temperature from 100 to 250 °C. Meanwhile, the amide peak at 1660 cm^−1^ is weak at 100 °C, which indicates that a significant portion of the amide group was involved in N-EP linkage formation. Unlike in Figure 4, at 200 °C, no re-appearance of the amide signal is observed in Figure 5. This may indicate that the regenerated amide upon linkage breaking is very short-lived and is imidized right after linkage breaking. Such a fast transition is not observed (reappearance of 1660 cm^−1^ peak) in Figure 5. This phenomenon of fast imidization can affect the pattern of linkage formation and EP homopolymerization and, consequently, affect the microstructure and physical property of the copolymer.

### 3.4. Morphology

**Figure 6:**
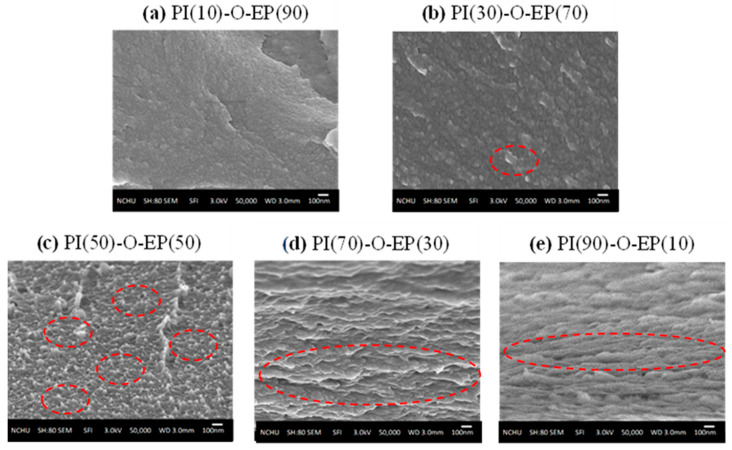
SEM of PI-O-EP (numbers in parenthesis are weight %); red circles mark typical microphase separation.

Microphase separation is an important phenomenon that is crucial to the physical properties of the copolymer [45]. In the literature [46,47,48], PI-EP blends were reported to exhibit phase separation. Ellis commented in his book [6] that during curing, mixing, and dissolution, the morphology of the copolymer may be affected. Figure 6 compares the SEM micrographs of the PI-O-EP copolymers at various PI/EP ratios (PI content 10% to 90%). In Figure 6a,b, EP is the continuous phase. In Figure 6d,e, PI is the continuous phase. Considering the large molecular weight difference between PI (ca. 8000) and EP (ca. 380), small molecules of EP are expected to be easily compatible with PI.

Therefore, in Figure 6a,b, the fracture surfaces are relatively smooth (less microphase separation). In Figure 6a, the dispersed PI is barely observable, whereas it is more observable in Figure 6b. In Figure 6d,e, the fracture surfaces are unsmooth, probably due to the microphase separation between PI and EP-homopolymer. In Figure 6c, since PI content is increased to 50%, the micrograph apparently shows a higher degree of microphase separation, and it seems that phase transition (or phase inversion) occurs at PI(50)-O-EP(50). For anticorrosion coating purposes, a smoothed matrix with little microphase separation would be more protective to the substrate. Thus, PI(10)-O-EP(90) and PI(30)-O-EP(70) would be of better choice in this regard. This will be further compared in the salt-spray test.

**Figure 7:**
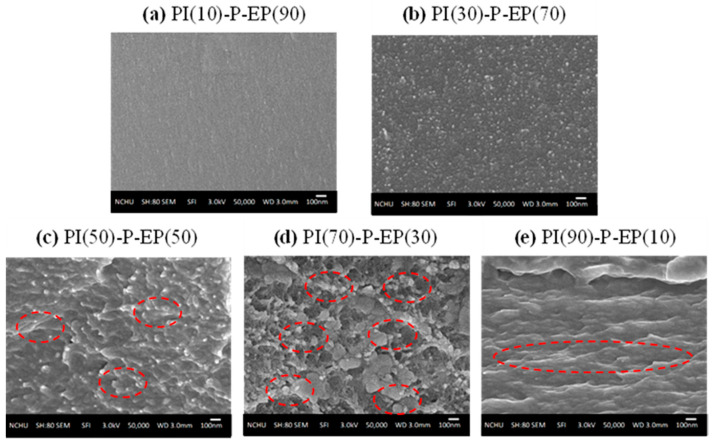
SEM of PI-P-EP (numbers in parenthesis are weight%); red circles mark typical microphase separation.

Figure 7 compares the SEM micrographs of PI-P-EP of various PI/EP ratios. The phase transition (or phase inversion) [39] shifts to PI(70)-P-EP(30) (compared to Figure 6). As stated above, the N-EP linkage breaking in PI-P-EP may take place later than that in PI-O-EP. In the latter case, the EP oligomer would have more opportunity to homopolymerize to larger blocks, which would reduce the miscibility between PI and EP. Thus, for PI-O-EP, microphase separation between PI and EP occurs at a lower PI content of 50%.

For PI-P-EP, as discussed in the FTIR section, fast imidization allows less opportunity for reappearance of the amide peak (1660 cm^−1^ peak at 200 °C). Consequently, with the decrease in opportunities for homopolymerization of EP, the homopolymerized EP blocks are relatively smaller than that in PI-O-EP. Therefore, the compatibility of PI in EP is better in PI-P-EP than in PI-O-EP.

### 3.5. IPN Structure

For PI-O-EPs, the terminal diamines first form PI-EP linkages, whereas for PI-P-EPs, the terminal dianhydrides form PI-EP linkages in a later stage. During curing and imidization, the major reactions involved in forming and breaking of the amide-EP linkage and imidization of the PI chain, as well as the homopolymerization of the released EP, are as follows:

From the PI-chain’s point of view, it is penetrating the EP network. For EP, some molecules were linked to the terminal diamines or dianhydrides of PI, while some (or the majority of the EP molecules) were homopolymerized to form EP-domain, which includes those EP molecules bonded to the terminal functional groups of PI.

According to Sperling [49], “Many interpenetrating networks (IPNs) exhibit dual phase continuity, which means that two or more polymers in the system form phases that are macroscopically continuous”. His comment provides a concise and clear description about IPNs. His narration of the IPNs’ structure provides a good basis for referencing the current case.

In the PI-EP networks, the PI polymer chains are linear and are cross-linked with the EP molecules at both ends of the PI chain. The molecular size of PI was estimated to be 8000 D, and that of EP is about 380 D. Such huge difference in size makes the two more miscible. However, due to the mutual miscibility and chemical linkages between PI and EP, the SEM of PI(10)-O-EP(90) (Figure 6a) and PI(10)-P-EP(90) (Figure 7a) found little or no microphase separation. Another reason for this phenomenon is the relative amounts of PI and EP. When the amount of the large-size PI increased, microphase separation becomes more and more apparent. Therefore, the structure of PI-EP in this study is closest to the definition of Semi-IPN by Sperling [47,49].

### 3.6. Mechanical Strength

For anticorrosion purposes, the coated film needs to be tough with some degree of flexibility. Otherwise, cracks could develop on the film due to factors like temperature and pressure variations. Table 2 compares the tensile modulus, tensile strength, and elongation at break of PI-O-EP and PI-P-EP with various PI content. As expected, the general trend is that all three measures increase with increasing PI content [49]. Table 2 reveals that chemically incorporating PI in the PI-EP copolymer gave the film improved mechanical properties.

Comparing these three mechanical properties (stiffness, brittleness, and ductility), for the same PI content, PI-P-EP is better than PI-O-EP (Table 2). Again, such contrast could be attributed to the observation that the extent of microphase separation is less in PI-P-EP than in PI-O-EP. The elongation at break of the two copolymers ranges from 0.85 to 9.35%, indicating that both have a certain degree of resilience that makes the coating film capable of coping with the metal’s expansion and contraction during temperature change.

The tensile strengths of PI-P-EP and PI-O-EP, at PI contents of 10%, (26, and 15 MPa) are comparable to the data (10 to 20 MPa) reported by Su et al. [48], who used polyamide and polyether amine as curing agents to improve the physical properties of EP resin.

For a good anticorrosion coating material, besides good adhesion property, the coating material must have good mechanical properties (such as toughness and some degree of ductility). Furthermore, the coating material should have no or minor microphase separation to prevent weak spots due to microphase separation from developing into cracks and providing passages for corrosive chemicals.

### 3.7. Thermal Properties

Table 3 compares the results of TGA analysis in terms of temperature at weight loss of 5% (Td(5%)) for PI-O-EP and PI-P-EP at various PI contents of the copolymer. Td(5%) of PI-O-EP and PI-P-EP share a common trend: Td increased with increasing PI content. This trend is in line with the expectation that PI would render improved thermal durability to the copolymer [50].

However, one set of data does not follow this Td(5%) trend; the Td(5%) of both PI(10)-O-EP(90) and PI(10)-P-EP(90) are higher than those of PI(50)-O-EP(50) and PI(70)-P-EP(30). This is coherent with the SEM results, which show that the microphase separations (of the 10/90 ratio) were minimal among all PI/EP ratio tested. Without microphase separation, PI was more homogeneously inter-penetrated with the EP matrix; therefore, its contribution in thermal stability was more profound than expected. Similarly, as stated above, PI-P-EP exhibited better compatibility of PI in EP than in PI-O-EP. Thus, for the same PI content, the Td(5%) of PI-P-EP are all higher than PI-O-EP.

### 3.8. Adhesion and Salt Spray Tests

A good anticorrosion coating must possess good adhesion properties with the coated material (most frequently metal). In this regard, EP has long been used in the metal coating industry [3]. For the copolymer under investigation to be a good candidate, it must possess as good, if not improved, adhesion properties as EP. Kim et al. [23] achieved excellent adhesion strength of the polyimide/EP joint using polyamic acid as both the adhesion-promoting layer and the curing agent.

The result of the cross cut adhesion test are listed in Table 3 [51]. With a high reputation of excellent thermal stability and toughness, PI’s application in metal coating has been limited due to its poor adhesivity. Table 3 shows that for various PI/EP ratios tested, the 10/90 set exhibited adhesion (100% adherence) that was as good as EP. The adhesion performances of the PI-EP copolymer are higher than 90% for PI content up to 70% (Table 3) [52,53]. If we compare PI-O-EP with PI-P-EP, the latter exhibits better adhesivity. This contrast could result from the aforementioned phenomena of reduced microphase separation, or, better yet, as a continuous and homogeneous one phase (Figure 6a and Figure 7a), as discussed in the SEM section.

Withstanding a serious salt spray test is prerequisite for designing an anticorrosion of metal coating material. In the above discussions about microphase separation, it is concluded that when we increase PI content to above 10 %, microphase separation becomes more apparent. Theoretically, phase separation indicates the existence of a region that is not continuous between the two microphases, which in turn is likely to be the weak spot of the material and might lead to structural failure under stress. Table 3 shows that the time before salt corrosion development is observed decreases with increasing PI content. In the meantime, Table 3 also shows that PI-P-EP outperformed PI-O-EP for all PI content studied. This is coherent with the above discussions about microphase separation.

## 4. Conclusions

In this study, the two polymers complemented each other in the PI-EP copolymer, namely with increased thermal durability, good ductility, and enhanced mechanical property (due to PI) and high adhesion strength (due to EP). In the early stage of the heating process (lower temperature at about 150 °C), EP curing was the dominant reaction (kinetic control), and at high imidization temperature (at about 200–250 °C), the dominant reaction was imidization (thermodynamic control). In between, the EP curing reaction was likely to be reversible, where some EP-amide linkages were broken and released amide function groups, which were then available for the competing imidization reaction to form PI. This is the key feature in the formation of IPN structure. The copolymerization reaction in this study follows the principles of kinetic vs. thermodynamic control of organic chemistry.

The linear PI chain and the PI-EP linkages formed an interpenetrating copolymer. Microphase separation was minimal at a PI content of 10%. Incorporating PI improved the mechanical properties of the PI-EP copolymer. At PI contents of 10 and 30%, the PI-EP’s adhesion strength was nearly the same as that of EP. Salt spray tests found that PI-EP provided the best protection at a PI content of 10%. In all perspectives investigated in this research, PI-P-EP is a better choice than PI-O-EP. In this study, PI is demonstrated to play an important role in polymeric hardener and forming an IPN structure with EP. The copolymer has high potential application in the field of anticorrosion.

This study merely covers limited information about the PI-EP copolymer. Process variables such as PI-EP ratio, molecular size, reaction time, and temperature control can be finetuned to meet requirements for specific applications.

## Figures and Tables

**Table 1:** Designed compositions of PI-O-EP and PI-P-EP composite copolymer.

System	ODA and PMDA Mole Ratio of PAA				
	ODA (mol)		PMDA (mol)		
PAA-ODA	0.24		0.23		
PAA-PMDA	0.24		0.25		
System	Weight ratio of PAA and EP				
PI-O-EP	10/90	30/70	50/50	70/30	90/10
PI-P-EP	10/90	30/70	50/50	70/30	90/10

**Table 2:** Results of mechanical tests for PI-EP films.

Copolymer System	PI/EP wt.%	Tensile Modulus (GPa)	Tensile Strength (Mpa)	Elongation at Break (%)
PI-O-EP	10/90	0.88	15	0.85
	30/70	1.70	25	1.79
	50/50	2.67	68	3.79
	70/30	2.68	80	4.77
	90/10	3.18	105	9.33
PI-P-EP	10/90	1.99	26	1.71
	30/70	3.16	82	8.24
	50/50	3.28	102	4.46
	70/30	3.37	115	3.91
	90/10	3.48	118	9.35

**Table 3:** T_d_(5%), cross cut adhesion test and salt spray test.

Copolymer System	PI/EP wt%	T_d_-5% (°C)	Number of Grids Not Flaked in 100 Grids	Salt Spry Test (h)
	10/90	355	100	1028
	30/70	367	98	697
PI-O-EP	50/50	346	96	361
	70/30	370	90	289
	90/10	442	77	91
	10/90	380	100	1368
	30/70	386	99	1088
PI-P-EP	50/50	395	98	577
	70/30	373	93	337
	90/10	461	84	111

## Data Availability

Not applicable.
